# Print-Collecting as a Hobby

**Published:** 1908-06-27

**Authors:** John S. Joule


### The Practitioner's Relaxations and Hobbies.

### PRINT-COLLECTING AS A HOBBY.

By JOHN S. JOULE, M.D.

It is not often in the voluminous papers, daily, weekly, and monthly, that one sees the devotees of hobbies or recreations, call these what we may, puffing up or defending their own special delectations. This shyness, for it is nothing else, is akin to the reluctance one has in airing the admirable qualities of those lovable creatures who are the objects of our special adoration. True, some hobbies seem to many very trivial, and if they be so, it is not unlikely they are harmless, and it is certain they afford pleasure and recreation to those who follow them. For the members, however, of a learned profession a hobby or recreation should have some feature in it of an intellectual kind. It should bring harmless pleasure in its pursuit; and it should not necessarily entail large, if indeed any, pecuniary burden. Having followed the recreation of collecting old prints for many years I can faithfully recommend this delightful pastime to the members of my profession. Nearly all men, and I should say all women, like pictures. Even daily papers do not rely solely on their descriptions of things and events. If these machine-made prints afford some pleasure to us all, what can be said for those admirable old engravings of a century and more ago? The human hand and eye and brain have brought them forth with marvellous skill; so that they have been a source of delight for ages, and will continue to be.

Medical men have been noted for their collections of prints. Many follow the pursuit now: and it is not to be wondered at, for I take it that a medical education in a special manner fits one to be a collector of prints. The would-be collector must, above all, be a good observer; if he fail in this he will have to pay for his errors. This hobby is also to be recommended in that it need not encroach on ordinary work. It can be laid on one side at the busy times, and taken up again in the quieter periods of practice. To those who are always busy with their professional or other work no hobby is needed. They have their reward. I do not envy them, and they do not need pity. Nor need the pursuit of print-collecting interfere in the least with the earnest and sympathetic way in which our profession carries out its duties. Yet there is nothing in a medical education or work to train the sense of the beautiful in art. Print-collecting supplies this want. For, however humble our resources, we can possess some nice prints, and learn from them the secret of their worth.

I doubt very much if anyone walking through a picture gallery really gets any knowledge of art worth speaking about. You might as reasonably expect to gain a knowledge of disease by simply walking along by the beds of a hospital. The ability to recognise a good print is, however, soon attained—for there are many good prints which, if not fashionable, are yet very admirable. Fashion does not seize upon everything that is good. Its appetite, though large, does not swallow up all that is good in prints, and the neglected of to-day may become the fashion of to-morrow. Print-collecting soon leads on to a better taste in the furnishing and decorating of our houses. Nor should the would-be-collector be discouraged by the high prices of some prints stated at print sales. As a rule, it is only the fashionable, and therefore high-priced, prints that are so advertised. Many admirable prints are sold at the same auctions for quite moderate sums. Very often I think the humble print surpasses in artistic worth its more aristocratic sister. The one is fashionable, the other not so—that is all. One costs hundreds of pounds, the other a few shillings. It is in the sale-rooms that a knowledge is best obtained of prints.

Let the collector use his own judgment. If he be a beginner, let him not buy at all for some time. Let him also pay more than one visit to the collection of old prints now on the walls of the British Museum, and left to the nation by that admirable collector the late Lord Cheylesmore. He will there see the very finest specimens of the art of engraving in mezzotint. Generally speaking, he should never buy a print unless it is in good condition, fine in impression, and fully satisfying his good sense of what is delightful, either in its work as a print, or in its beauty. Mistakes he will make—all do—and what he thinks fine to-day may a year hence not quite satisfy him. Let him then improve his collection, weeding out what he considers imperfect. I feel sure that he who collects as a hobby and from the love of the things he does collect will have no reason, financially, to regret the pursuit.

In following out the hobby of print-collecting a certain amount of leisure is necessary, although it need not be continuous. The pursuit can be left alone for weeks or months and taken up again. The collector in or near London has an advantage over one more remote, for there is no market in the world so large and varied in this as in other branches of works of art. Given, then, some leisure, and above all a love of pictures, print-collecting may be followed by almost anybody with moderate means. A friend of mine with a salary of not more than £200 a year was a collector of prints for over thirty years. His business occupation was very monotonous—sitting in an office the greater part of the day—and the enjoyment he took in searching for his prints I feel sure added to the prolongation of his life. For myself I can say that the forming of my collection during the last twenty years has been a source of the greatest interest and pleasure. Unlike many hobbies, it has few vexations; it can be carried on to old age, and so be a source of interest at a time when sustained work is not for us.

I think it not unlikely that many brother practitioners may have a desire to make a collection of old prints, but have been deterred from following it out from want of guidance; or from the fear that the pursuit was too complicated. The reports of sales in the daily papers may add to the confusion, in that only high-priced prints are there recorded, and the conclusion may be drawn that it is hopeless to buy such expensive things. These expensive prints, although fine and beautiful, are expensive because the fashionable rich have made them so. But there are still many fine prints of men equal, at any rate, in artistic merit to the fashionable prints of grand ladies, and to be bought for comparatively small sums. Prints neglected to-day may become the fashion ten years hence. Engravings " in line " were the fashion fifty years ago; to-day they are quite neglected, except in a certain class of French prints. To-day mezzotint portraits of ladies and a few noted men—together with stipple or Bartolozzi prints, especially in colour—are the fashion. It is curious to note how collectors incline to different types of prints: some will only look at etchings, others at stipple and mezzotints, and others again at nothing but coloured prints, and others pass from one line of collecting to another, and, like myself, finish up with the mezzotint portraits. It is in mezzotint engraving that the Englishman has surpassed all other competitors. No other nation nas produced such a fine line of workers as ours of a century ago. Bartolozzi at the head of the stipple engravers was a foreigner, but in mezzotint our countrymen, J. R. Smith, Valentine Green, the two Watsons, and many others, excelled all others. Much, however, may be said in favour of all the forms of engraving, and many very beautiful examples exist of them all.

I think it best in making a collection not to mix the types of prints, but to adhere to one or the other of them. Having decided on this, the collector should learn thoroughly to know a fine print from an ordinary one. It is only in the early and sharp impressions of a plate that a print can be judged. Many an ordinary-looking print, which would scarcely be noticed, may prove in its fine and early state to be one of great beauty. A stipple print of Miss Bingham or of Countess Spencer may, if weak, be a dear print at £5, and, if very fine, cheap at £50. Some prints, although old and genuine, are so rubbed or pale as to be worth nothing at all; yet one sees such prints sold at auctions. They are bought by people who have not learned to know a good print from a bad one. Reprints—and their name is legion—are even worse when sold as old ones. They are put in old frames with old, dirty paper at the backs, even old nails in the back boards; and I have seen the glass inside dusted over so as to obscure the shy beauty beneath. This being so, how is the beginner to buy safely? He should buy at first under the guidance of some honest dealer and expert. Happily there are several, and they are not difficult to find. Mistakes will be made, but the true collector is not discouraged by them. He becomes more wary as he gets older, and there is no such thing as senile decay in the collecting world.

No one, I think, will deny that a fine portrait of a beautiful woman after Reynolds, Romney or Hoppner is a more decorative object, be it a print or a picture, than that of a man. But this class of print is becoming exceedingly scarce and costly. Portraits of men are far more easily obtained. They are engraved by the same hands as the women's portraits, and certainly no less skill has been bestowed on them. Men's portraits are being more and more appreciated, as is seen by their increased market prices. The points to be observed in the selection of them are in the main that they be portraits of men of note in politics, literature, etc., or the portrait should be " decorative "—that is, it should be a pleasing picture. It should be in good condition: not rubbed or worn, but clear and distinct. And the earlier the state of the print the better. It is a good plan to have one or two very fine examples on the walls, so as to familiarise the eye and serve as a guide. A good collection of prints, however, can only be made by educating oneself in the subject. In London, at any rate, the opportunities are at hand for the making of such a collection. And be the collection fine or modest in its quality, it will, I am sure, afford a most pleasant recreation in the bringing of it together. Medical men, even lawyers and parsons, are not as a rule a very happy looking lot of men when seen together. Is it because they are the slaves of one pursuit? Do doctors carry their cases in their faces? If so, perhaps it were better to have some mitigating influence, even that of a humble print-collector.

